# Supplementary material for: Are carbon emissions trading and green financial instruments synergistic? -Comprehensive quantitative research based on content analysis
Source: PLoS One. 2024 Mar 7;19(3):e0298601. doi: 10.1371/journal.pone.0298601 (PMC10919601; doi:10.1371/journal.pone.0298601)
Supplement: S1 Data — (ZIP) [file pone.0298601.s001.zip › Data/地级市Z_F工作报告.zip!/╔╜╬≈/╜·│╟╩╨/└┤╫╘╣1⁄2╓┌║┼í╛┬φ┐╦╩2╛▌═°í┐_2013.docx]

各位代表：

现在，我代表市人民政府向大会作工作报告，请予审议，并请市政协委员和列席人员提出意见。

一、2012年工作回顾

过去的一年，在中共晋城市委的正确领导下，在市人大的有效监督和市政协的大力支持下，市政府团结带领全市人民群众，认真贯彻落实党的十七届六中全会和十八大精神，围绕打造山西经济重要增长极和山西新兴产业重要支撑基地的目标，着力增投资、壮产业、创优势、惠民生，总体呈现出经济较快增长、民生持续改善、社会和谐稳定的良好态势。全市生产总值登上千亿台阶，达到1011.6亿元，同比增长11%；固定资产投资完成655亿元，增长29.9%；财政总收入突破200亿大关，达到213.5亿元，增长17.4%；公共财政预算收入完成82.9亿元，增长22.1%；规模以上工业增加值增长14.6%；社会消费品零售总额完成260.8亿元，增长16.2%；外贸进出口总额完成12.35亿美元，增长8.9%；城镇居民人均可支配收入完成22565元，增长12.1%；农民人均纯收入完成8037元，增长14.1%。市六届人大二次会议确定的各项目标任务基本实现,“方便晋城”十大工程全部完成。被评为山西省2012年度目标责任考核优秀市。

——产业转型取得新成效。项目建设和重点工程全力推进，省级重点工程完成率134.5%，居全省第一。传统产业进一步巩固提升。煤炭产业，巩固兼并重组成果，新转产验收和联合试运转矿井14座，产能规模达975万吨/年。煤层气产业，地面抽采井达到5500口，年抽采能力达到40亿立方米。中国联盛、新奥集团、晋煤天煜等煤层气液化项目建成运行。一批电力、煤化工项目开工建设。非煤主导产业成长壮大。高新技术产业快速崛起，装备制造业规模不断扩大。富士康A区苹果手机精密机构件、金鼎煤机等标杆项目加紧推进。商贸物流业快速发展,苏宁电器、必胜客、凤展新时代广场正式营业，太行明珠游乐城、皇城相府城市综合体等项目加紧建设。旅游产业蓬勃发展，“晋善晋美、精彩晋城”提升了知名度和美誉度。全市旅游总收入、接待入境旅游者、旅游外汇收入、国内旅游收入增幅均居全省第一。与此同时，积极推进非煤主导产业集群化发展，形成了县域经济错位发展、差异竞争、优势互动的良好势头。

——对外开放迈出新步伐。深度对接上海、富士康、华润、晋煤，招商引资持续升温。与上海及长三角地区签约项目48个，其中落地25个，总投资172亿元。金匠工业园区完成一期采空区治理和场地平整。华润兰花制药项目落地。深入实施融入中原战略，加大旅游景点建设和营销力度。联合河南、河北等省的20多个城市，共同打造“太行山文化旅游”精品。全力推进综改试验区建设，编制了转型综改行动方案，完成了“一市两园”总体规划，开工建设标杆项目18个。全市招商引资实际到位资金561亿元，增幅居全省第一。

——“三农”工作实现新突破。认真落实中央强农惠农富农政策，农民收入稳步增长。粮食总产量19.5亿斤，创历史最高水平。生猪出栏达到258.4万头，全省第一。大力发展“一村一品、一县一业”，扎实推进农业产业化，新建现代农业科技园区30个，农民专业合作社发展到2980家。300个重点推进村完成了规划编制、“四化四改”和“五个一工程”。开展了首届“晋城最美乡村”评选活动。重点扶持146个贫困村，生产生活条件明显改善，新的“五个全覆盖”工程全部超额完成。

——城市品质得到新提升。强力推进城市建设，实施了一批城建重点工程。改造提升了凤台街、文昌街两条主干道，取消了群众呼吁强烈的西上庄收费站，打通了兰花北路、红星街东西延伸段、文昌街西段、建设北路、客运东站环路等断头路，新增城市道路7公里。“三馆合一”主体完工。加快推进城市水系建设，任庄水库调水工程完工，滨河南路完成路基建设。开工建设保障性住房11560套，占省定任务的181%；竣工5291套，占省定任务的130%。市区经济适用住房被评为“国家康居示范工程”。

——生态环境又有新改善。强力推进污染减排，关闭落后产能及生产设施企业10个，取缔市区分散燃煤供热锅炉102台。完成污染减排重点项目38个。丹河人工湿地三期工程通水试运行。市区空气质量二级以上天数353天，稳定达到国家二级标准，综合污染指数控制在1.72。加大节能降耗力度，实施节能项目52个。完成村庄绿化300个，造林12.1万亩。被评为“全国国土绿化突出贡献单位”，荣获“国际花园城市”称号。

——社会事业取得新进展。爱物学校、汇仟小学主体完工，中等专业学校一期工程和凤城中学项目顺利推进，新建标准化幼儿园15所。在全省率先实现了十二年免学费教育，为农村寄宿制学校配备校车76辆。加快推进晋城大医院建设，正式启用市、县两级医院远程医疗会诊系统，投资6个亿加强县乡村三级医疗基础设施建设。城镇医疗保险标准全省第一，工伤、失业、生育、城乡居民养老保险标准居全省前列。积极推进就业和全民创业,新增城镇就业岗位4.12万个，城镇登记失业率1.8%，被国务院授予“全国创业先进城市”。城乡低保、五保供养保障标准名列全省前茅。群众性文化、体育活动蓬勃发展。“文化低保”工程覆盖面扩大，“周末大剧场”、“百姓大舞台”等活动深得人心。统计、地震、气象、人防、治超、民族宗教、妇女儿童、人口计生、档案史志、外事侨务、老龄、残疾人、红十字、慈善救助等社会事业，都取得了新的成绩。

——和谐稳定呈现新局面。深入推进精神文明和民主法治建设。自觉接受人大监督，认真听取各民主党派、工商联和无党派人士意见。办理人大代表建议意见267件、政协提案305件。坚持依法行政，加强政风行风建设，连续8年被省政府表彰为“政风行风评议先进市”。创新社会管理机制，城市社区网格化管理试点工作圆满完成。深入开展“严打”整治斗争，强化信访和社会矛盾排查调处工作，“平安晋城”建设成效显著,在全省群众安全感和满意度民意测评中名列第一。全民国防教育更加深入，国防后备力量建设质量大幅提升。安全生产形势持续平稳，各类事故起数和死亡人数均低于省控指标。

各位代表，这些成绩的取得，是认真贯彻执行党中央、国务院和省委、省政府一系列方针政策的结果，是中共晋城市委统揽全局、正确领导的结果，是市人大、市政协有效监督、鼎力支持的结果，是全市人民辛勤工作、共同努力的结果。在这里，我代表市人民政府，向全市人民，向驻市解放军指战员、武警官兵和公安干警，向各民主党派、工商联、各人民团体和社会各界人士，向所有关心和支持晋城发展的同志们、朋友们，向为本届政府打下良好工作基础的王清宪市长，表示衷心的感谢和崇高的敬意！

同时，我们也要清醒地认识到：发展不充分、不协调、不科学仍然是当前的主要矛盾。产业结构一煤独大，新兴产业和高新技术产业发展不足。发展方式粗放，节能减排和安全生产压力较大，科技创新驱动支撑作用明显不足。农民增收难度加大，社会事业发展和民生改善与人民群众的期盼还有较大差距。发展环境、政府职能和干部作风等都需要切实转变。对此，我们要高度重视，认真加以解决。

二、2013年工作安排

2013年，是全面贯彻落实党的十八大精神的开局之年，是实施“十二五”规划承上启下的关键一年。综观国内外形势，我们仍然处于可以大有作为的重要战略机遇期。党的十八大为我们指明了前进的方向，山西综改试验区和中原经济区的政策效应形成了加快发展的强劲态势。特别是“一争三快两率先”的战略部署，极大地鼓舞了全市干部群众干事创业的热情。同时我们也要看到，世界经济复苏艰难曲折，国内经济增长趋缓，能源原材料产能过剩价格下滑，还有很多制约发展的体制机制障碍等等。我们既要坚定信心、抢抓机遇、顺势而为、乘势而上，又要增强机遇意识、忧患意识、积极应对、赢得主动。

今年政府工作的总体要求是：深入贯彻落实党的十八大精神，坚持以科学发展观为指导，突出主题主线，坚持好中求进，按照市委“一争三快两率先”的战略部署，着力改革开放，着力项目建设，着力民生改善，在加快产业转型上有新突破、在加快统筹城乡上有新作为、在加快建设美丽晋城上有新成效，为率先全面建成小康社会、率先走出资源型地区科学发展新路奠定坚实基础。

综合考虑各方面的因素，今年我市经济社会发展的主要预期目标是：全市生产总值增长11%以上，固定资产投资增长24%以上，财政总收入和公共财政预算收入分别增长14%，城镇居民人均可支配收入增长12%，农村居民人均可支配收入增长12%以上，社会消费品零售总额增长15.5%。

实现上述目标，重点要做好八方面工作：

（一）以“一争三快两率先”统揽各项工作，全力推进改革开放。

坚决破除影响和制约科学发展的思想观念和体制机制，营造更具活力、更加开放的发展环境，使各类市场主体释放出发展的巨大潜力、活力和持久动力。

解放思想。思路决定出路，观念决定发展。没有思想的更新就没有发展的突破，没有思想的突破就没有发展的跨越，没有思想的解放就没有发展的率先。在新的发展阶段，我们必须清醒认识自己的差距，认真审视存在的不足，坚决摒弃“小富即安、知足常乐”的“满、慢”心态。牢固树立争先三晋、挺进中原、先行先试、勇于担当的豪情壮志。用思想的解放引领行动的率先，推动“一争三快两率先”战略目标的顺利实现。以壮士断腕的决心推进改革，构建强大的发展正能量。以思想的大解放，推动晋城的大开放、大发展，赢得发展主动权。

深化改革。发展的难题就是改革的取向，要用改革的办法破解发展中的难题。围绕新一轮政府机构改革和转变政府职能，深化行政审批制度改革，审批事项要能减则减、能放则放、能快则快。提高行政效能，切实做到人民政府为人民服务、为基层服务、为企业服务、为项目服务。重点推进煤层气管理体制改革、市政建设建管体制改革、投融资体制改革、园区体制改革、户籍和社会保障制度改革。同时继续深化财政体制改革、文化体制改革和土地供给创新等，并要务求实效。

扩大开放。围绕招商引资、招才引智，深化和重点地区、重点企业全方位合作对接。以更加开放的理念，在更大空间、更高层面上优化配置晋城资源。加快与中原经济区的沟通融合，推进区域一体化发展。要利用资源优势，形成产业链关系，加强文化旅游合作，共同打造“太行山文化旅游”精品。积极推进焦作—晋城城际铁路、新乡—陵川高速公路、阳城—济源高速公路的立项和建设。要在产业转移、交通体系、商贸物流等方面全方位开放、多层次协作，力争把我市建设成为中原地区人流、物流、信息流汇集区。积极推进晋城海关、国检机构和综合保税区建设，为进一步对外开放提供保障。

先行先试。要按照全省转型综改“四大任务”要求，攻坚克难、先行先试，加快综改标杆项目、重点园区、先行试点建设。要把巴公镇作为综改试点区，扩权强镇，园镇一体化，为全市率先走出资源型地区科学发展新路进行积极的探索。同时要抓好“一市两县”、“一市两园”、“一县一企”和“一市一板块”、“一县一任务”等试点工作。抓好民政等“厅市合作共建试点”，力争在先行先试中有新突破。加快建立健全转型综改试验的评价、考核体系，鼓励各级各部门解放思想、先行先试。

（二）加快产业转型升级，率先走出资源型地区科学发展新路。

坚持改造提升传统产业与培育壮大新兴产业联动，承接产业转移和增强内生动力并举，加快推进产业结构调整和集聚集群发展，着力提升产业竞争力，提高经济发展的质量和效益。

改造提升传统产业。坚持“以煤为基”，做好煤炭这篇大文章，为产业转型奠定基础争取时间空间。加快矿井整合改造，提高规模化、集约化、现代化水平。完成矿井投资59亿元，建设转产验收和联合试运转矿井19座，形成有效产能1500万吨/年。积极应对煤炭市场变化，努力保障煤炭经济稳健运行。加快煤制油、煤转电等发展步伐。重点推进晋煤天溪、晋煤华昱、煤电一体化、热电联产、低热值煤发电等项目建设。加快 “气化晋城”步伐，加快煤层气的综合开发利用，到2015年建成国家级沁水盆地煤层气产业化基地。

不断壮大非煤产业。坚持“多元发展”，加快产业转型。以富士康为龙头，打造“高新技术产业集聚区”，加快金匠园区富士康项目建设，完成富士康A区工程建设并投产运行，支持唐一、硕阳、融高等企业做大做强，引进扶持一批配套服务的科技型企业。大力发展装备制造产业，加快清慧、路宝等汽配、铸造企业规模化、集群化发展，完成金鼎煤机一期工程，打造全国有一定影响的“装备制造产业集聚区”。积极发展现代服务业，加快金融财富广场、红星美凯龙、兰花国际商城、豪德二期等城市商业综合体建设，打造“区域性商贸物流中心”。做大做强文化旅游产业，壮大吉利尔潞绸、珏山二十八星宿动漫等文化产业，支持晋氏织造、晋陶陶瓷等文化创意企业发展，规划开发书院文化，打造“区域性文化产业集群”。整合全市资源禀赋，推动历史、文化、自然与旅游的深度融合。加大城市形象宣传力度和皇城相府、王莽岭等重点景区品质提升，办好第二届“太行山国际文化旅游节”，打造“中原地区重要的休闲度假旅游目的地”。

加快推进园区建设。园区经济是转型发展的重要载体。要尽快研究出台《加快工业园区建设实施意见》，完善规划，理顺体制，建立健全高效率的运行机制和开发模式。要加大政府投入，加快园区的基础设施和公共服务平台建设。要明确准入门槛，提高引进项目质量，提高园区土地利用率。努力实现政策向园区倾斜、项目向园区布局、企业向园区集中、产业在园区循环、人才向园区流动，充分发挥工业园区资源共享、产业集群、企业集聚和辐射带动作用。

大力发展实体经济。研究出台支持实体经济具体办法，支持冶铸、汽配、陶瓷、蚕桑等传统优势行业兼并重组，形成一批具有自主创新能力的大企业、大集团。支持企业产品开发、品牌创建、市场开拓的力度，培育更多销售收入超亿元企业。帮助餐饮酒店业克服困难，渡过难关。加快建设融资担保、创业辅导、企业孵化、人才培训、技术支撑等公共服务平台，加大小微企业扶持力度。深化市场资源要素配置改革，激活民间资本，确保各类投资主体公平待遇。

扎实开展“项目推进年”活动。认真落实“六位一体”工作机制，今年招商引资要完成签约项目总投资1600亿元，项目实际到位资金600亿元，落地资金510亿元，开工项目总投资完成371亿元，确保固定资产投资的快速增长。省市重点工程要优化环境，加强调度，落实责任，确保年度目标圆满完成。要下功夫引进一批效益型、高科技、标杆性的大项目、好项目。加大项目推进力度，切实做到待批项目抓前期，已批项目抓开工，开工项目抓进度，竣工项目抓投产，投产项目抓达效。

（三）加快生态绿色发展，持续打造美丽晋城。

坚持把生态文明建设放在更加突出的位置，全面落实资源节约和环境保护基本国策，加强生态系统建设，推进绿色、循环、低碳、可持续发展，努力建设美丽晋城。

加大环境保护力度。坚持预防为主、源头治理。深入开展绿色生态工程，加快生态环境综合整治。加强对“丹、沁”流域排污企业的环境监管，推动企业污水深度处理和中水回用工程建设。加快丹河流域生态恢复治理步伐，规划建设丹河两岸城市生态景观走廊。大力开展饮用水水源保护区工程，保障人民群众饮水安全。开工建设市污水处理厂二期工程和餐厨垃圾、粪便无害化综合处理厂，启动一化、中石油仓库搬迁。实施农村清洁工程，开展好“十镇百村”建设。逐步建立雾霾天气预警机制，积极实施环境空气质量新标准，有效提升空气质量监测能力，为美丽晋城增光添彩。

狠抓节能减排。开展国家低碳城市试点工作，认真编制低碳发展规划和温室气体排放清单，实现高碳产业，低碳发展。强力推进节能减排，实施排污权有偿交易。加大对高耗能、高污染企业和项目的监管力度，确保各项节能减排指标实现预期目标。严格执行环评、能评制度，严禁高耗能、高排放项目审批。继续加速淘汰落后产能，指导和督促企业按期淘汰。推进交通运输节能，开展绿色建筑行动，大力推进绿色建筑试点示范。

大力发展循环经济。加快国家循环经济标准化试点城市建设步伐，按照“减量化、再利用、资源化”的原则，以提高资源产出效率为目标，推进生产、流通、消费各个环节循环经济发展。开展在农业、工业、建筑等重点领域推进清洁生产示范，从源头和全过程控制污染物产生和排放，降低资源消耗。加强煤矸石、粉煤灰等固废资源综合利用及废水、废气、废渣循环利用，形成具有明显晋城区域特点的资源综合利用产业链。倡导文明、节约、绿色、低碳消费理念，推动形成绿色生活方式和消费模式。

加强生态林业建设。围绕创建“国家森林城市”目标，加快造林绿化步伐。规划启动环城森林公园建设。重点实施“两山”造林、“两网”绿化等六大林业工程，突出抓好环城绿化、村庄绿化、通道绿化、矿区绿化。全市新增造林面积20万亩。推进护林防火生物隔离带建设，切实做到防火与造林并重。力争森林覆盖率每年继续提高一个百分点。

各位代表，建设集约高效、宜居适度、山清水秀的美丽晋城，是全市人民的共同愿望。我们必须以对子孙后代和未来负责的态度，竭尽全力让晋城的城市更加靓丽，乡村更加优美，风景更加怡人！

（四）加快实施“四位一体”战略，促进城乡一体化发展。

大力实施中心城市、县城、产业片区和中心镇“四位一体”的城镇化发展战略，努力构建以中心城市为龙头、以县城为骨干、以产业片区和中心镇为纽带的新型城镇发展格局。

加快中心城市建设步伐。以迎接建市30周年为契机，围绕打造中原经济区最具核心竞争力城市的目标，全面提升中心城市综合承载力和辐射带动力。东南拓展，西北改造，中心提质。“拓展”以金村新区、金匠区为重点；“改造”以西北片区、兰花路片区、北石店城市化改造和城中村改造为重点；“提质”以白马寺森林公园和完善路网为重点，铺开景西路北段延伸、文博路北段、太岳路、207国道改线等17条道路工程，加快贯通主城区道路，畅通与北石店区、金村区、南村区之间的快速通道。重点抓好皇城相府城市综合体、城市会展中心、文化艺术中心、商务大厦等地标建筑。加快花园头河景观水系主体工程、城市公园绿地、新植物园建设。配套建设北石店、金匠污水处理厂等10项市政基础设施。

加快“大县城”建设。以扩大规模、增强实力、完善功能、体现特色为基本路径，加强高平、阳城、陵川、沁水次中心城市建设，积极引导生产要素、优势资源向大县城集中。完善县城基础设施和公共服务设施，增强县城对县域经济的带动作用和吸纳集聚人口的能力，充分发挥经济、文化、人口相对集中的优势和沿企、沿河、沿路相对便利的条件，形成与中心城市优势互补的功能区。

加快产业片区建设。围绕巴公装备制造工业园、金匠高新技术产业工业园、高平煤焦化工业园、北留周村煤电化工业园、沁水新能源产业工业园和高平新能源科技创新园，以产业为支撑，以园区为载体，通过产业集聚带动人口集聚，通过工业化带动城镇化。集中打造北留—周村等六大产业片区。在加快片区基础设施建设、夯实产业支撑的同时，启动建设一批对周边城镇具有辐射作用的社会公共服务设施，以及供水、供热、治污、垃圾处理等基础设施，提前规划公租房或限价商品房建设，提高片区的承载力，为片区项目落地、人员入住提供保障。“十二五”期间，六个片区规划城镇人口规模达到10万人。

加快中心镇建设。中心镇是联络城乡一体发展的纽带。要扩权强镇，统筹城镇与新农村建设、土地流转与规模化经营，带动周边乡村人口向中心镇集聚。突出抓好沁河流域、丹河流域、环城高速“两河一路”城镇组群发展。重点推进巴公、马村等20个中心镇建设。着力提升中心镇公共服务能力，实现“一个标准化中学、一个标准化卫生院、一个集贸市场、一个集中式供水站、一个文化体育活动场所”的“五个一”标准，逐步形成集聚功能明显、各具特色、统筹发展的中心镇格局。同时，要尊重农民意愿，稳步开展试点，促进农村人口向农村新型社区集中，推动农民就近城镇化。

（五）加快发展现代农业，不断增加农民收入。

始终把“三农”问题作为重中之重，认真落实各项强农惠农富农政策，有效促进农民增收，巩固和发展农业农村工作大好形势。

多措并举增加农民收入。加强农村劳动力转移培训工作，提高农民的务工收入。挖掘农业内部增收潜力，提高农民经营性收入。加大支农资金整合力度，增加对农民的直接补贴，切实保障农民的转移性收入。完善征地补偿机制，鼓励农村土地向农业园区流转，引导农民进入园区就业，实现一份土地资源带来投资、工资两份收入的目标，不断增加农民财产性收入。

做大做强特色农业产业基地。以“一村一品”、“一县一业”为主攻方向，新建“一村一品”特色专业村200个，“一村一品”特色乡镇10个。巩固发展一批畜牧、蚕桑、蔬菜、食用菌、小杂粮、中药材等为代表的特色农业基地；建设一批集生态农业、循环农业、观光农业、资本农业为一体的综合性示范园区；扶持一批市场开拓能力强、经营规模大、辐射带动面广的农业产业化龙头企业；培养一批创新技能人才和农业实用技术人才，以农业产业化发展带动农民增收致富。

抓好农村基础设施建设。加快“井”字型骨干供水网、农田水利灌溉网、城乡一体化饮水安全网建设，突出抓好郭壁供水改扩建等七项重点水源工程的开工建设。加大中低产田改造，建设高标准农田。加强耕地保护，加大土地开发力度，增加耕地面积。积极引导农民按照“依法、自愿、有偿”的原则流转土地，实现土地规模化经营，为农业机械化和农业现代化打下基础。

加大新农村建设和扶贫开发力度。以中心镇为重点，提升农村公共服务水平，推动农民生产生活方式转变。重点抓好100个新农村建设和10个新农村示范片建设，抓好小城镇、乡（镇）政府所在地及中心村建设。巩固提升“五个全覆盖”成果。加大150个贫困村产业发展扶持力度，增强贫困村自我发展能力。继续做好领导干部下乡住村、市县单位结对帮扶、党员干部帮贫到户工作。

各位代表，“三农”工作事关农民切身利益，事关社会和谐稳定，事关全面建成小康社会目标实现。我们要更加重视、切实抓好“三农”工作，让农业更强，让农村更美，让农民更富！

（六）加快发展社会事业，着力保障和改善民生。

继续加大民生投入，在学有所教、劳有所得、病有所医、老有所养、住有所居上取得新进展，努力顺应广大人民群众的新向往、新追求、新期待，让人民过上更加美好的生活。

积极促进就业创业。全面引深国家级创业型城市创建工作，健全城乡就业体系，以创业带动就业，培训帮助就业，援助扶持就业，鼓励多形式就业，创造更多岗位，推动实现更高质量就业。改善就业环境，保护合法权益，构建和谐劳动关系。全年城镇新增就业3万人，完成城镇失业人员再就业培训和新成长劳动力培训1万人。

全面增强社会保障能力。坚持全覆盖、保基本、多层次、可持续的方针，推进城镇职工和城乡居民养老保险衔接、城镇职工医保与居民医保、新农合衔接。提高城镇居民医保、新农合补助标准和实际报销水平。落实农民工融入城市和被征地农民社会保障实施办法，进一步扩大社会保障覆盖面。实行惠农补助一卡通。试行启动社会养老机构建设、运营政府补助制度和购买服务制度。加快发展普惠型社会福利和社会服务体系建设，确保我市社会福利水平位居全省前列。

推动教育均衡协调发展。加大投入，科学布局，努力办好人民满意的教育，使得不同群体的孩子能够方便上学、充分就学、快乐学习。扩大学前教育资源。加快推进城乡义务教育均衡发展，实施义务教育免课本费全覆盖，完成全市教育城域网“校校通”，完成县级义务教育学校标准化建设。逐步改善市区薄弱学校办学条件，持续增加教育资源总量。完成爱物学校和汇仟小学建设，加快中等专业学校和凤城中学项目建设，解决好班容量过大和优质教育资源配置不均衡等问题。大力发展和提升现代职业教育, 支持特殊教育发展，促进民办教育规范健康发展。坚持建校和引校两手抓，力争今年晋城学院有实质性进展。继续深化教育综合改革，加强教师队伍建设，有效提升教育教学质量。逐步解决农村中小学音体美教师短缺问题。

提高人民健康水平。深化医药卫生体制改革，扩大县级公立医院改革试点，同步推进医药卫生一体化综合改革。加快推进晋城大医院建设，启动市人民医院外科楼建设和市中医院前期工作。加强基层卫生服务标准化建设。完善新农合制度，参合率保持在98%以上，重大疾病保障范围由6类扩大到20类。实施好国家基本公共卫生服务项目，防控重大传染病暴发流行。大力加强医疗卫生人才培训和引进，切实提高医疗水平。

加快发展科技、文化、体育等社会事业。继续加大科技投入，引进高层次人才，提高自主创新能力。切实加强人口和计划生育工作，稳定低生育水平，提高出生人口素质，促进人口长期均衡发展。围绕创建国家公共文化服务体系建设示范区，继续加大文化工作扶持力度，加强文艺精品创作，推动文化大发展大繁荣。广泛开展全民健身运动，促进群众体育和竞技体育全面发展，办好第五届市运会。大力发展老龄、残疾人、慈善、红十字事业，继续做好统计、地震、气象、人防、对台、档案史志、民族宗教、外事侨务、文物保护、妇女儿童等各项工作。

（七）加快社会管理创新，维护和谐稳定发展大局。

加强民主法治建设。认真实施“六五”普法，扎实推进法治政府建设。认真执行人大及其常委会的决议、决定，自觉接受人大、政协和社会各界的监督，主动接受人民和舆论监督。认真办理人大代表建议和政协委员提案，广泛听取民主党派、无党派人士、人民团体意见。让人民更有效监督政府，使政府更好服务人民，决不允许庸懒散奢，决不允许弄虚作假，决不允许损害群众利益！

加强精神文明建设。积极践行社会主义核心价值观，凝聚起实现伟大“中国梦”、“晋城梦”的精神力量。着力推进公民和未成年人思想道德建设。深入推进全国文明城市创建活动，充分发挥道德模范的示范作用。广泛开展群众性精神文明创建活动，巩固发展市县乡村“四位一体”的文明创建格局，大力弘扬晋城精神，人人争做“晋城好人”，提高全民文明素质，培育良好社会风尚，塑造文明晋城新形象。

加强和创新社会管理。加强社区建设，健全基层社会服务网格化管理体系，提高基层社会管理服务效能。完善信访工作和矛盾调处机制，加强社会组织的培育，切实解决好事关人民群众切身利益的问题。加强食品药品安全监管，保障人民群众饮食用药安全。继续加强国防动员、国防教育和国防后备力量建设，促进军民融合发展。进一步加强应急管理，健全突发事件应急体系。做好治超和防灾减灾工作。深入开展平安建设，严厉打击各类违法犯罪活动，不断增强群众安全感。进一步完善社会治安防控体系，维护社会和谐稳定。

加强安全生产工作。要牢固树立发展是第一要务，安全生产也是第一要务，抓好发展是政绩，抓好安全生产也是政绩的理念。要严格责任，进一步强化政府的安全监管责任和企业的安全生产主体责任，做到责任到人、责任到岗、责任到秒。要严格监管，做到强化制度执行、制度落地，严格落实安全生产规章制度。要夯实基础，强化安全生产“三同时”管理，做到没有安全设计不开工，没有安全验收不生产。要严肃纪律，用铁的手段，严厉打击各种违法违规行为，有效防范和遏制重特大事故发生，确保安全生产持续平稳。

（八）加快政府职能转变，努力提升科学发展能力。

改进作风、狠抓落实是政府工作的主要任务，加快政府职能转变、提升科学发展能力是加强政府自身建设的主要内容。要时刻牢记权力是人民赋予的，要时刻牢记我们是人民的公仆，要时刻牢记“勤政廉政，心中有老百姓”，要时刻牢记最好的工作是只争朝夕，最好的落实是一抓到底，最好的公信是说到做到。

提高履职能力，创建学习型政府。深入创建学习型机关，全面提升公务员素质。要注重方针政策的学习，提高掌握政策、运用政策、用足政策、用活政策的水平；注重信息化、城镇化、社会管理等领域的学习，提高解决复杂矛盾的水平；注重现代科技、现代金融等专业知识的学习，提高研判国际国内科技和产业发展最新动态的水平；特别要加强市场经济知识的学习，提高运用市场的思路和办法，解决改革发展难题的水平。

转变政府职能，建设服务型政府。正确处理政府与市场的关系，推动政府职能向创造良好环境、提供优质公共服务、维护社会公平正义转变，为人民群众和市场主体提供优质便捷高效服务。围绕开展“学党章、守纪律、转作风”专题教育活动，深入实际、深入基层、深入群众调查研究，切实解决实际问题。要狠抓各项工作落实，做到“具体化、责任化、目标化”，各项工作都要在全省争先竞位、争创一流。

坚持依法行政，建设法治型政府。进一步增强法治观念，严格按照法定程序行使权力。建立健全科学民主决策机制，完善重大公共政策群众参与制度，确保各项决策体现民意、符合实际。严格规范行政执法，完善行政复议机制。深入开展民主评议政风行风工作，推进政府信息公开，加大政务公开力度，确保权力在阳光下运行。深化行政监管执法机制改革，强化行政执法监督，确保行政服务和执法的规范化、制度化、标准化，不断提高依法行政水平。

严格廉洁从政，建设清廉型政府。全面落实党风廉政建设责任制，完善惩治和预防腐败体系，努力从源头上防治腐败。加强监察、审计监督，规范权力运行，坚决惩处权力寻租、权钱交易等违法违规行为。厉行勤俭节约，反对铺张浪费，严格控制行政经费支出，集中财力办大事。今年全市公用经费要实现零增长，“三公”经费削减百分之二十。

在做好以上各项工作的同时，还要回应人民群众关切，办好十件实事：一是建设标准化幼儿园50所；二是解决特困户危房改造及移民搬迁2897户；三是完成清洁村庄建设1200个；四是完成行政村街道亮化1538个；五是市区新增供热面积140万平米；六是启动气化晋城，新增城乡清洁能源用户4万户；七是新开工建设城镇保障性住房1.5万套；八是城乡居民基础养老金提高到80元；九是新增公交车100台；十是建设城市公共自行车交通系统。

各位代表，“空谈误国、实干兴邦”，唯有脚踏实地、扎实苦干才能实现既定目标。让我们紧密团结在以习近平同志为核心的党中央周围，在中共晋城市委的坚强领导下，解放思想，开拓进取，齐心协力，真抓实干，奋力开创我市转型跨越发展新局面，为实现“一争三快两率先”战略目标，谱写晋城人民美好生活新篇章而努力奋斗！
